# Evaluating scale effects of topographic variables in landslide susceptibility models using GIS-based machine learning techniques

**DOI:** 10.1038/s41598-019-48773-2

**Published:** 2019-08-23

**Authors:** Kuan-Tsung Chang, Abdelaziz Merghadi, Ali P. Yunus, Binh Thai Pham, Jie Dou

**Affiliations:** 1 Department of Civil Engineering and Environmental Informatics, Minghsin University of Science and Technology, Hsin-Chu, 304 Taiwan; 2 Research Laboratory of Sedimentary Environment, Mineral and Water resources of Eastern Algeria, Larbi Tebessi University, Tebessa, Algeria; 3 State Key Laboratory of Geo-hazard Prevention and Geo-environment Protection, Chengdu University of Technology, Chengdu, 610059 China; 4 Institute of Research and Development, Duy Tan University, Da Nang, 550000 Vietnam; 5 Key Laboratory of Geological Hazards on Three Gorges Reservoir Area Ministry of Education, China Three Gorges University, Yichang, China; 6 Nagaoka University of Technology, 1603-1 Kami-Tomioka, Nagaoka Japan

**Keywords:** Natural hazards, Geomorphology, Civil engineering

## Abstract

The quality of digital elevation models (DEMs), as well as their spatial resolution, are important issues in geomorphic studies. However, their influence on landslide susceptibility mapping (LSM) remains poorly constrained. This work determined the scale dependency of DEM-derived geomorphometric factors in LSM using a 5 m LiDAR DEM, LiDAR resampled 30 m DEM, and a 30 m ASTER DEM. To verify the validity of our approach, we first compiled an inventory map comprising of 267 landslides for Sihjhong watershed, Taiwan, from 2004 to 2014. Twelve landslide causative factors were then generated from the DEMs and ancillary data. Afterward, popular statistical and machine learning techniques, namely, logistic regression (LR), random forest (RF), and support vector machine (SVM) were implemented to produce the LSM. The accuracies of models were evaluated by overall accuracy, kappa index and the receiver operating characteristic curve indicators. The highest accuracy was attained from the resampled 30 m LiDAR DEM derivatives, indicating a fine-resolution topographic data does not necessarily achieve the best performance. Additionally, RF attained superior performance between the three presented models. Our findings could contribute to opt for an appropriate DEM resolution for mapping landslide hazard in vulnerable areas.

## Introduction

Globally, landslides are one of the most devastating of geo-hazards that impose serious threats to human life and economic conditions by the never-ending socio-economic burdens^1^. With the recent changes associated with the unplanned urban expansions and severe climatic extremes, landslides are expected to increase dramatically due to the heavy rainfall and the serious infrastructure constructions in mountainous areas^2,3^. To reduce the economic burden and human losses, it is helpful to delineate and identify potential landslide-prone areas. In this regard, landslide susceptibility mapping (LSM) is regarded as a useful tool in disaster management and mitigation^4–7^. The susceptibility zonation maps can be the first step towards a complete risk assessment that assist authorities and decision-makers for initiating appropriate mitigation measures. Over the past few decades, LSM techniques were extensively developed and implemented by several researchers, and each technique has proven to have its own sole merits and demerits^6,8,9^.

Landslide susceptibility expresses the likelihood of a landslide event occurring in a given area based on local terrain conditions. LSM partitions the geographical surface into zones of varying grades of stability based on evaluating the consequence of the probability toward landsliding generated using the causative factors estimated to induce the instability^10–13^. Landslide susceptibility mapping (LSM) plays a significant role in risk mitigation, especially in introducing counter-measures aimed at decreasing the risks associated with landslides^14,15^. Moreover, LSM could be engaged to depict locations of unknown landslides in the future, support with emergency-decisions, and mitigate future hazardous events.

For landslide susceptibility evaluation, GIS has proven to be a powerful tool due to its capability of handling a variety of spatial data, their processing capability, and easiness in the decision-making process^9,16,17^. Over the years, landslide susceptibility assessment topic has gained significant attention of many scholars owing to its direct consequences on the people life. Numerous studies have been carried out for landslide susceptibility assessment around the world in the current decade using a wide variety of techniques^9,18^. Several of them employed the relationship between the landslide causative factors and landslide occurrence through the spatial data analysis^8,13^. Such relationships can be categorized in terms of rankings or weight. In this context, such data-driven methods can be classified into two distinct categories: qualitative and quantitative^13,19^. The former are rather subjective^20^. On the other hand, the latter are based on statistics, and with the development of computer systems and GIS tools, these models have become more prevalent than the qualitative methods^21^. Quantitative methods such as logistic regression, fuzzy logic, certainty factor, and information value approaches are useful for problem-solving and have been successfully used in different scientific fields, such as engineering, and hazard evaluation applications. Very recently, numerous machine-learning (ML) techniques have been applied for different fields, owing to their robustness in handling large complicated data^8,22–25^. These methods include Artificial Neural Networks (ANN), Decision Trees (DT), Support Vector Machine (SVM) and Random Forests (RF) were comparatively new in the field of landslide research. Despite different statistics involved, their terminologies, and computation capability, all of the aforementioned methodologies are largely based upon the following assumptions^20^; (i) past is the key to the future; (ii) factors involving the landsliding are spatially linked and therefore could be used in predictive functions; (iii) future events will likely happens in similar conditions.

Apart from the statistical and computational aspects, the accuracies of the susceptibility model depend upon the quality of spatial data and the choice of relevant causative factors. Diverse intrinsic and extrinsic factors are cast-off to analyze LSM. The typical factors that can be derived from a DEM and other sources which influence the landslides are known through several past research. For example, the review of ‘statistically-based landslide susceptibility models’ by Reichenbach *et al*.^20^ grouped the influencing factors into five categories; (i) morphological, (ii) geological, (iii) land cover, (iv) hydrological, and (v) others. Süzen and Kaya^26^, listed about 18 causative factors in the triggering mechanism of landslides. However, in any given situation, some of these factors may be important whilst others are irrelevant^18^. These factors come from different sources, and their quality varies widely. Thus, the landslide evaluation exclusively based on a digital elevation model (DEM) has been conducted assuming that topography reflects other causative factors such as hydrology and land use. The availability of global DEMs and recent advances in DEM acquisition techniques encourage this approach.

Accurate topographic input comes from high-quality DEMs, along with the geological conditions are usually necessary for producing accurate susceptibility products^18^. Generally, DEM’s produced using interpolation of contours from a topographic map, radar-based Shuttle Radar Topographic Mission (SRTM) DEM, and stereo-optical derived Advanced Space-Borne Thermal Emission Radiometer (ASTER) DEM are used for susceptibility analysis if no high-resolution DEM is available for the studied region. They come with varying spatial resolution; 10 m – 90 m. A coarser DEM describes the terrain less accurately, resulting in the propagation of error on to the secondary derivatives such as slope, aspect, and curvature, etc.^27^.

While the scale effects of landslide causative factors, particularly topographic variables derived from DEM are well-known issues in geomorphology, only a few studies have attempted the potential effects it may have on susceptibility models. For example, Guzzetti and others^28^ suggested the use of multiple resolution-DEMs for testing and to opt the best performed one in final susceptibility mapping. However, with the increasing availability of very high-resolution DEMs derived from LiDAR and UAV images, researchers tend to use 1–5 m DEM’s in their modeling part expecting that a finest DEM can describe more detailed topography. Paudel *et al*.^29^ studies, however, argued that the smallest-scale variability does not well represent the physical processes because the local topography does not resemble the processes of controlling landslide initiation. On the other hand, Tian *et al*.^30^ by analyzing 5–190 m DEMs, indicated that the optimal resolution often depends on the chosen size of the study area. Catani *et al*.^31^ coined the term Mapping Unit Resolution (MUR) to define the raster resolution and performed the scale effects out at six different MUR (10–500 m). Their results are in line with^29^, where the finer resolutions are found less accurate. Nevertheless, performing sensitivity analysis was recommended when LSM results are utilized for planning and protection purposes in a given area. Conclusive answers for identifying the optimal scale for global reach, therefore needs further investigation, especially for hazard assessments. A poor understanding of scale effect may inadvertently promote frequent use of high-resolution DEMs, thus demanding substantial computational requirements. Because disaster management and mitigation require quick responses, timely interventions are necessary. Therefore, a special focus was given in this work to address the scale dependency in detail.

With these objectives in mind, the present work aims at: (i) producing a rich analysis of the effect scale dependency in landslide assessment; (ii) demonstrating the appropriateness of certainty factor model in selecting significant influencing factors; and (iii) address the landslide susceptibility issue for the study area by benefiting from multiple machine learning models. To achieve the aforementioned objectives, we employed an integrated approach comprising three varying resolution elevation models, certainty factor for investigating the relationship between correlated factors and landslide occurrence, and the widely applicable statistical and machine learning models such as Logistic Regression (LR), Random Forest (RF), and Support Vector Machine (SVM) for producing the susceptibility maps. All analyses were conducted in the programming environment R (3.6.0). The source code of this research is publicly made available online (https://github.com/aminevsaziz/lsm_in_Sihjhong_basin) to ensure results reproducibility. ArcGIS (10.4) and SAGA (4.0.1) were used for compilation and visualization of factor and susceptibility maps.

The general idea of selecting LR, RF, and SVM in our analysis is that LR is the most popular susceptibility model^19^, whereas RF and SVM are the most promising ones^9,13^. While LR is simple, straightforward, and highly interpretable, however, it cannot solve non-linear problems. RF, on the other hand, produces a more accurate and robust prediction, but is less descriptive^9^. SVM though delivers a unique solution for complex problems with its kernel tricks, but the kernel-specific parameter selection is a complex process. A combination of learning models increases the overall understanding of the issue, but the computational requirements vary. Therefore, we also aimed to quantify the average time required to train and test each of these popular models in view of mitigation preparedness.

## Study Area and Data Used

### Overview of the study area

**Figure 1:**
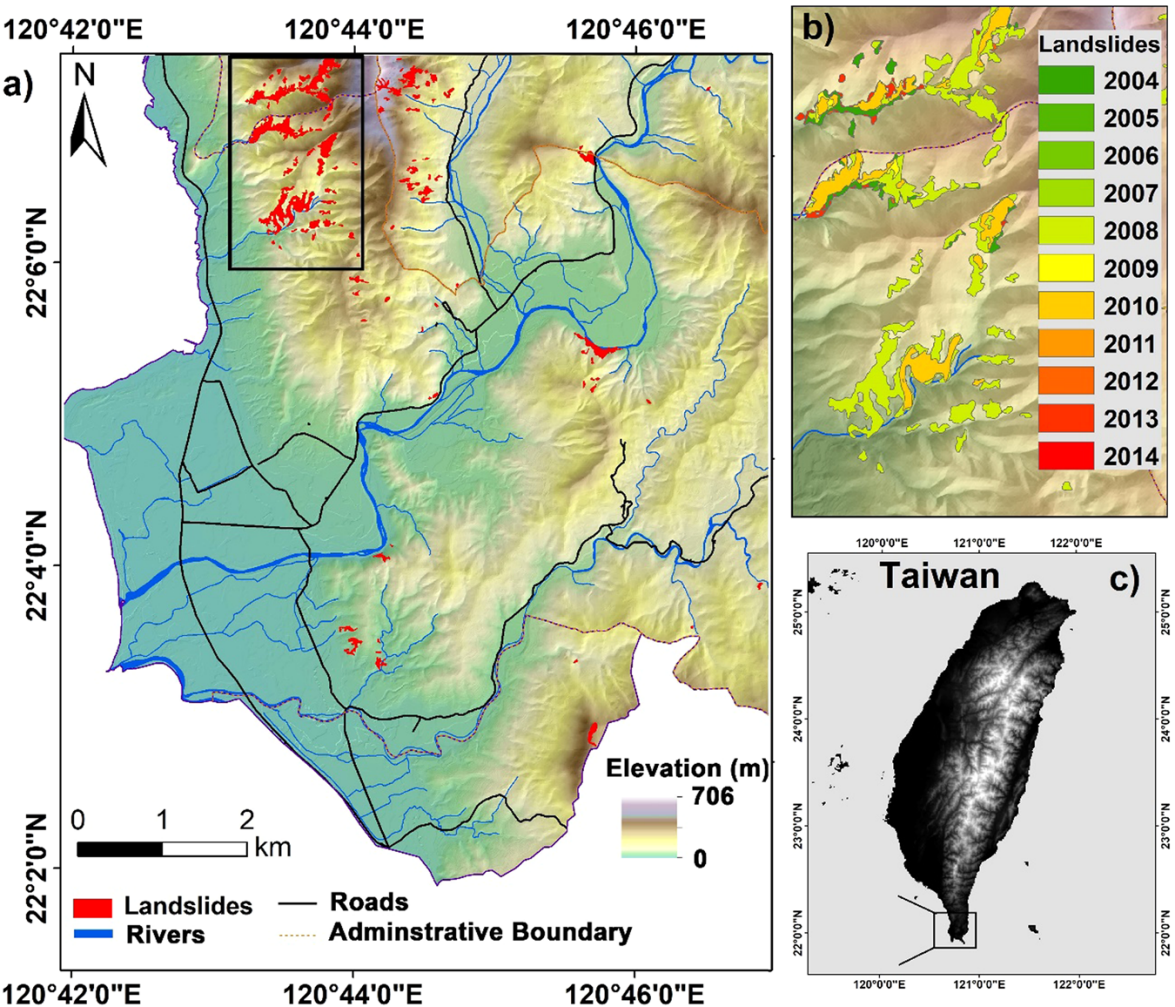
(**a**) The location of the case study area showing the landslide inventory, major roads and river network, (**b**) inset showing the details of multi-temporal landslide polygons overlying the DEM, (**c**) overview map showing the study region within Taiwan.

Taiwan has a land area of 36,000 m^2^, 26.68% of which is covered by plains, whereas 27.31% is hilly and 46.01% is mountainous. According to the statistics of the National Fire Agency (NFA), many natural disaster events are occurring in Taiwan, include typhoons, flooding, earthquakes, torrential rainfall, windstorms, and landslides. The selected study area-Sihjhong watershed is located in the Hengchun Peninsula in the southern part of Taiwan (Fig. 1). Because of sustained economic growth and land development, the steep terrain in this region has undergone frequent modification in land use pattern. Windward portion of the selected watershed in the recent past has suffered from multiple landslides (Fig. 1b) triggered by heavy rainfall during Pacific typhoon seasons. On average, about five typhoons are expected to affect the Island nation a year. In recent years, it is aggravated by global climate change. Rainfall is plenty in the peninsula and annual accumulated rainfall can be reached up to 3600 mm. The altitude of the study area varies from 0 m to 700 m with a mean of 110 m. Moderately gentle to steep hills and mountains are typical of the Hengchun Peninsula. On the west, the study area is bounded by the South China Sea with flat long coastal plains. The average and maximum slope derived from a 5 m LiDAR DEM are 15° and 66° respectively. Geologically, the study area is composed of thick sedimentary strata. The most dominating lithological unit in the Sihjhong area is Shale with alteration sequence. A detailed description of individual lithologic types is provided in the data section. From a disaster perspective, Sihjhong is an important case area with multiple hazards from typhoons (e.g., flood and landslides) in the sight which may aggravate with extreme climate^32^. Therefore, performing landslide susceptibility analysis is key for providing baseline information to practitioners and lawmakers^6,11^.

### Data used

**Figure 2:**
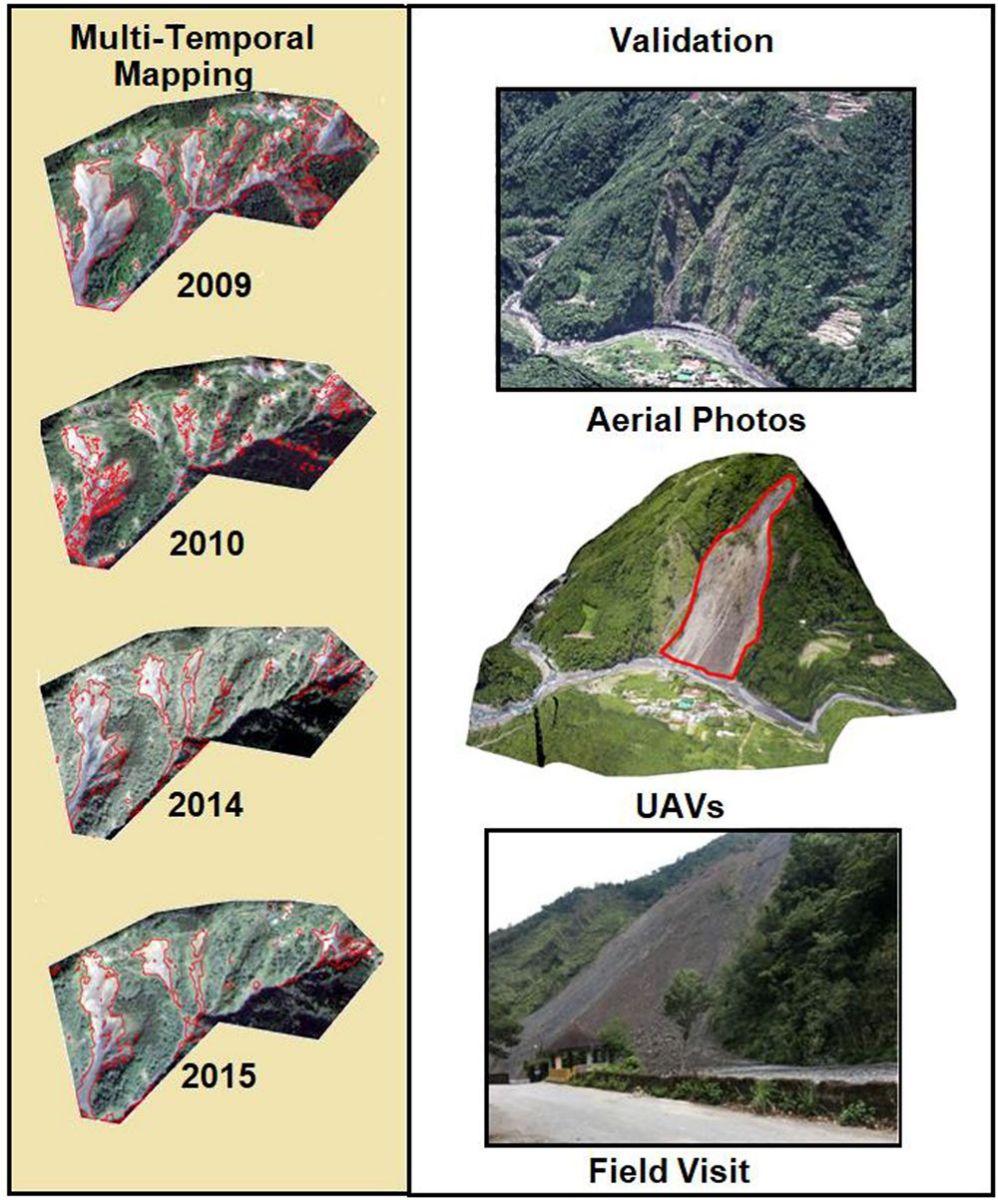
Examples of the landslides inventory maps constructed by dynamic time series analysis in the study area (Left Formosat-2 satellite images acquired from National Space Organization, Taiwan as part of projects funded to the first author. Right side images are downloaded from https://www.nlsc.gov.tw/, under open government data license https://www. data.gov.tw/license).

The multi-temporal landslide inventory database for the study area from 2004 to 2014 was portrayed in Fig. 1. Figure 2 shows examples of a landslide inventory map prepared from dynamic time-series image analysis carried for the study area. This dataset was downloaded from the NGIS Data Warehouse and Web Service Platform (TGOS Portal) developed by the Information Center, Ministry of Interior in Taiwan. The landslide inventory was created by interpreting Formosat-2 satellite data and an expert landslide and shaded area delineation system (ELSADS). The accuracy of landslide inventory has been carefully validated manually with the help of aerial images at 25 cm spatial resolution. The overall accuracy of this inventory was tested previously and found to be 98%^32^. The number and area statistics of landslides and typhoon details for each landslide inventory in the study area from 2004 to 2014 is illustrated in Table 1. Many landslides occurred in 2008 because of short duration and high-intensity rainfall.

**Table 1:** Statistics of multi-year landslide inventory (2004 to 2014) for the study area.

Year	Landslide number	Min. Landslide area (m^2^)	Max. Landslide area (m^2^)	Total Landslide area (m^2^)	Typhoon number	Max. 24 h Rainfall (mm)
2004	29	1252	49892	231620	9	1107
2005	12	1512	18164	56848	7	789
2006	3	1228	2628	5104	7	205
2007	9	1188	17774	53910	6	64.7
2008	111	270	103471	731855	6	1390
2009	8	1357	19910	49295	4	193
2010	29	116	32933	172510	5	629
2011	14	35	11426	56603	5	702
2012	13	1087	14563	57282	7	590
2013	26	41	54736	221620	6	820
2014	13	497	16359	39250	3	559
Total	267	8583	341856	1675897	65	6984

**Figure 3:**
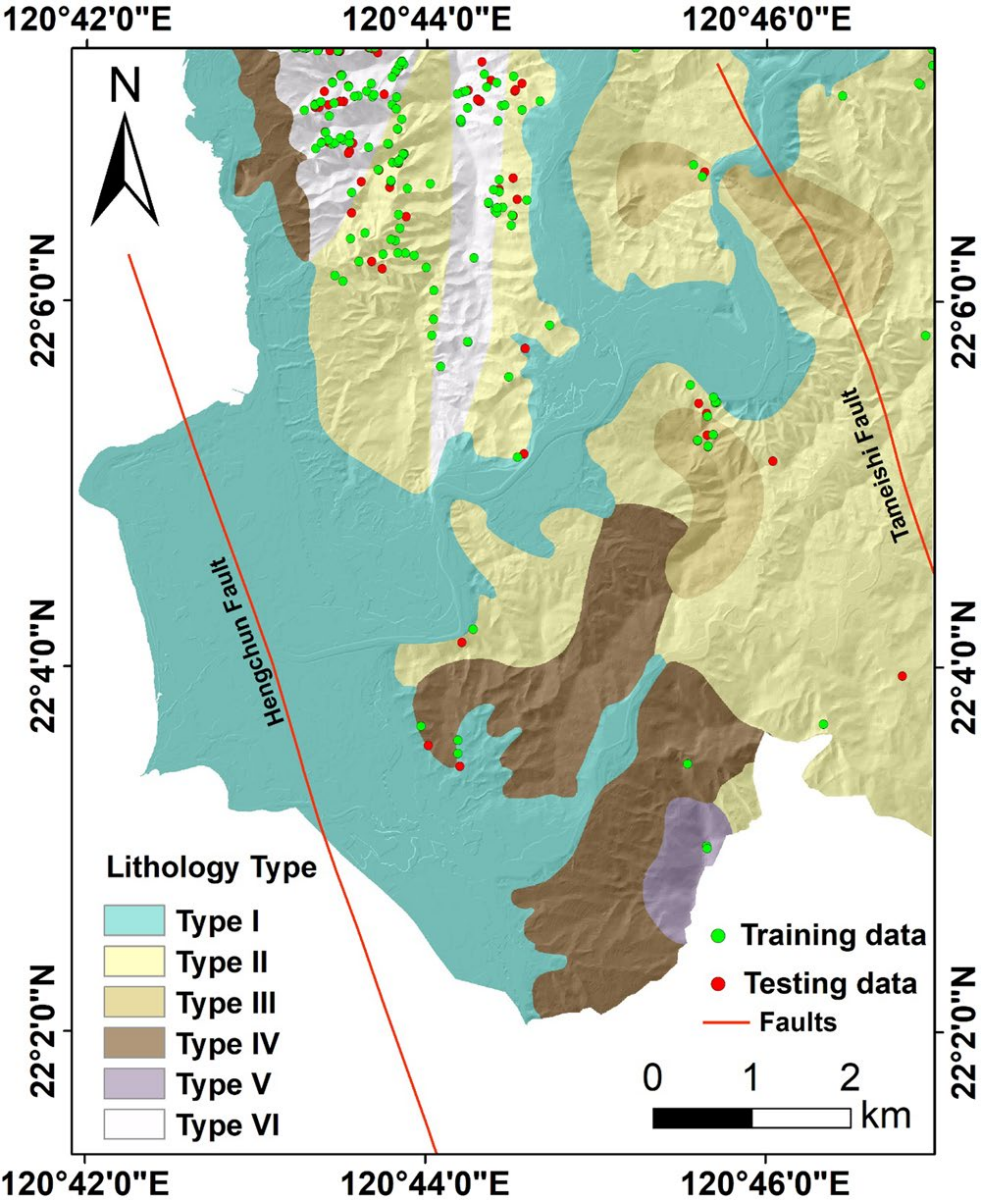
Geology map of the study area (Scale 1:50000) depicting six types of lithology.

The causative factors influencing the spatial distribution of landslides have been extensively explained in literature^6,13,19^. A general summary of these studies suggests that selection of the landslide predisposing factors in a given case should take into account: (i) the characteristics of the study area, (ii) the landslide type, (iii) scale of the analysis, and (iv) the data availability^13,14,21^. The causative factors selection in this research was based on the aforementioned summarization concerning spatial relationships between landslide occurrence and causative factors comprising topography, hydrology, tectonics, geology, and geomorphology^9,14,19^. Tectonic factor (distance to fault) was later discarded because faults are not corresponding with the landslides identified in Fig. 3. Moreover, the triggering mechanism for our landslide inventory was attributed to rainfall alone^33^.

After careful assessment, a total of twelve landslide causative factors were finally selected for this case study, i.e., elevation, slope angle, slope aspect, total curvature, plan curvature, profile curvature, terrain position index (*TPI*), terrain roughness index (*TRI*), distance from the road, distance from drainage networks, rainfall, and lithology. All twelve causative factors were processed and analyzed with the assistance of SAGA and ArcGIS® software. The first eight causative factors were derived from three different digital elevation models (DEM).

DEM is a digital grid form of representation for the terrain’s surface. DEM can be created from various technologies, such as Terrestrial Surveying, Aerial Photogrammetry, Light Detection and Ranging (LiDAR), Interferometric Synthetic Aperture Radar (InSAR). The common applications of DEMs include geomorphometric feature extraction, hydrological modeling, geo-hazard inventory, light-of-sight analysis, and landscape modeling and ecosystem management, etc. High-quality DEMs are required for precise applications. For this study, we have used two kinds of DEM’s: the first one is a LiDAR-derived 5-meter DEM (hereafter termed as 5 m LiDAR DEM) derived from investigation results of changes in surface topography and environmental geology caused by Typhoon Morakot, happened on August 2009 from Central Geological Survey in 2013. After field verification, the overall geometric accuracy found between 0.5 and 1.0 m.

The second kind is the ASTER Global Digital Elevation Model with 30-meter resolution (hereafter named 30 m ASTER DEM) is a joint product developed and made available to the public by the Ministry of Economy, Trade, and Industry (METI) of Japan and the United States National Aeronautics and Space Administration (NASA). It can be available free of charge to users worldwide from the Land Processes Distributed Active Archive Center or shortly LP DAAC (https://lpdaac.usgs.gov/products/astgtmv002/). The vertical accuracy of ASTER GDEM version 2 had been revealed a standard deviation is 5.9~12.7 meters. (https://asterweb.jpl.nasa.gov/gdem.asp). Additionally, we resampled the 5 m LiDAR DEM into 30 m resolution using bilinear interpolation technique to have a comparison with the 30 ASTER DEM. The source of the road and hydrology network map used in this study is obtained from a digital map of the traffic network produced by the Ministry of Transportation and Communications. Lithology data is digitized from a 1:50000 Geology map produced by the Central Geological Survey (Fig. 3). There are six lithology types contained in the area including Gravel, sand and clay (Type I), Shale and thin alternation of sandstone and shale with thick-bedded sandstone and conglomerate lentil (Type II), Sandy conglomerate (Type III), Mudstone and various exotic blocks (Type IV), Thick-bedded sandstone, interbedded sandstone and shale (Type V), and Thick-bedded sandstone intercalated with conglomerate (Type VI).

## Methods

### Implemented models

We employed three popular machine learning algorithms to map landslide susceptibilities. While logistic regression (LR) is a parametric machine learning algorithm (learning model that summarizes data with a set of parameters of fixed size - no matter how much data we input at a parametric model, it won’t change its mind); both support vector machine (SVM) and random forest (RF) are non-parametric models (algorithms that do not make strong assumptions about the form of the mapping function; also the complexity grows as the number of training samples increases)^19,34^. Among these two non-parametric models, RF does not need any real hyperparameters to tune, whereas SVM requires tuning for the right kernel, regularization penalties, and the slack variable^13,35^. Detailed description and computation of each ML algorithm are provided in the following sections.

#### Logistic regression

Logistic Regression is a popular statistical modeling method which has been applied widely in many problems such as gene selection in cancer classification and crime analysis^18,36^. In landslide susceptibility analysis, the LR has also used popularly in many case areas^19,37^. In the LR, the main mathematical concept is to use the logit-the natural logarithm of an odds ratio, which is expressed as follows:

1 $$logit(prob)=\{\begin{array}{c}(\frac{prob}{1-prob})\\ {\alpha }_{o}+{\alpha }_{1}{x}_{1}+\ldots +{\alpha }_{n}{x}_{n}={\alpha }_{o}+\mathop{\sum }\limits_{i=1}^{n}{\alpha }_{i}{x}_{i}\end{array}$$

where: n is the number of the variables used, *α*_*o*_ means the intercept, and *α*_*i*_ are defined as the coefficients related with the explained variables *x*_*i*_, and *prob* means the probability of a landslide occurrence which is a nonlinear function of *x*_*i*_ is expressed as follows:

2 $$Prob\,(x)=\{\begin{array}{c}\frac{1}{1+{e}^{-logit(Prob(x))}}\\ \frac{1}{1+{e}^{-({\alpha }_{o}+{\sum }_{i=1}^{n}{\alpha }_{i}{x}_{i})}}\end{array}$$

#### Support vector machine

Introduced by Vapnik^38^, Support Vector Machine (SVM) is a well-known unsupervised learning machine learning method which has been applied successfully and effectively in landslide susceptibility mapping^34,39^. The main concept of the SVM is to apply the linear model to carry out the nonlinear class boundaries by nonlinear mapping the input vectors into the new high-dimensional feature space where the optimal separating hyperplane is built to separate output classes for classification. More detail, the optimal separating hyperplane is the maximum margin hyperplane, which offers the maximum separation between the output classes, and the training samples which are closest to this hyperplane called support vectors. In the linearly separable problem, the optimal separating hyperplane of binary decision classes can be computed as follows^40^:

3 $$y={w}_{o}+{w}_{i}{x}_{i}$$

where y is defined as the outcome class, x_i_ means the input variables, and w_i_ mean the weights which determine the hyperplane.

#### Random forest

Random Forest (RF) is an effective ensemble classifier, which constructs multiple decision trees for classification utilizing a subset of variables randomly selected^41^. It is a machine learning technique as well, which has been used to solve a lot of real-world problems such as monitoring of land cover, predicting protein-protein interactions, predicting disease risks^9,35^. In landslide prediction, the RF has also been applied in several types of research. In literature, the RF is a popular method with high performance as it has several advantages such as (1) It is a non-parametric nature-based method, (2) it is able to determine the importance of variables used, (3) it provides an algorithm to estimate the missing values, and it is flexible for the analysis of classification, regression and unsupervised learning^42^.

In the RF, one subset of the predictor variables are utilized to construct each tree, and the number of trees (n_tree_) and the number of the predictors used to build each tree (m_try_) can be different which depend on the dataset. Using the RF, each tree is constructed from a bootstrap sample of primary training dataset used to estimate the robust error with the testing dataset expressed as follows:

4 $$MSE={n}^{-1}\mathop{\sum }\limits_{i=1}^{n}({t}_{i}-\overline{{t}_{i}})$$

where *MSE* means mean square error calculated during constructing the classification trees, n is the number of out of bag observation in each tree, $\overline{{t}_{i}\,}\,$is defined as the average of whole out of bag predictions^43^. Percentage of the explained variable is calculated as follows:

5 $${V}_{ex}=1-\frac{MSE}{{V}_{z}}$$

where: *V*_*z*_ means the total variation of the response variable. At last, the outcome of the RF is one single prediction that is the mean of all aggregations.

### Certainty factor (CF)

The certainty factor (CF) model is an approach for handling uncertainty in rule-based systems, which has been broadly used in expert system shell field, additionally, to medical diagnosis studies^15^. The CF model is one of the probable favorability functions to solve the problem of incorporating heterogeneous data^44^. The universal theory function is expressed as:

6 $$CF=\{\begin{array}{c}\frac{P{P}_{a}-P{P}_{s}}{P{P}_{a}\ast (1-P{P}_{s})}\,if\,P{P}_{a}\ge P{P}_{s}\\ \frac{P{P}_{a}-P{P}_{s}}{P{P}_{s}\ast (1-P{P}_{a})}\,if\,P{P}_{a} < P{P}_{s}\end{array}$$

where *PP*_*a*_ is the conditional probability (CP) of owning a number of landslide events happen in class *a* and *PP*_*s*_ is the prior probability (PP) of owning a total number of landslide events in the case study area. The value of *PP*_*s*_ this study is computed to be 0.012.

The range of CF value varies [−1, 1]. Positive values denote an increasing certainty in landslide occurrence; negative values imply a decrease in the certainty. A CF value near 0 shows that the prior probability is near to the conditional probability, and thus, it is difficult to determine the certainty of landslide occurrence^15^. The favorability values are acquired by overlapping landslide inventory maps and each data layer and calculating the landslide frequency. The CF model provides a rank measure of certainty in forecasting landslides. The relationship between the landslide sites and used causative factors had been analyzed in this study.

### Construction of the geospatial database for the training and the validation dataset

The input dataset obtained from the geospatial database of this experimental research was fed directly into the required models without extra encoding (i.e., dummying or numerically decoding of categorical variables) because the selected models handle efficiently diverse space variables (i.e., numeric and categorical). Also, it is critical to understand that the input dataset is not only for training the models. In the absence of an independent testing dataset, a common approach is to estimate the predictive performance based on resampling the original data. These strategies divide the data into training sets and a testing sets, while ranging in complexity from the popular simple holdout split to K-fold cross-validation, Monte-Carlo K-fold cross-validation, bootstrap resampling^45^. They can be used efficiently for models selection, accuracy assessment, and hyperparameters tuning^3,13^.

In our study, the input dataset was randomly split into two sets (training and testing datasets) by 70:30 ratios, and then the training set was innerly resampled using ten k-folds cross-validations. The implemented resampling approach is considered as the golden standard for machine learning, because they are found effective as it reduces the split randomness that comes with test-train split strategy, which allows the input dataset to be used for three different purposes: (1) tuning models hyperparameters, (2) to train models with this subset using after optimal parameters are found, and (3) models validation, assessment, and comparison.

### Model configuration and implementation

Some models (i.e., RF and SVM) require a fine-tuning for its hyperparameters on which the model performance depends. Usually, such feat is achieved by manual tuning using techniques such as grid search, random search, and even gradient-based optimization. However, such techniques have proven to be suboptimal at best, considering the fact that manually exploring the resulting combinatorial space of parameter settings is quite tedious and tends to lead to unsatisfactory results. Moreover, the obtained optimal hyperparameters cannot be reproduced to a certain degree, and that is because of such techniques rely on “Trial and Error” experimenting, which depends on analyzing that learning curves and decide that best learning path. This drawback is so critical especially if the modeling experiment involves complex experiments with a fair amount of data to process and for that fact, we opted for a State-of-the-art algorithm so-called sequential model-based optimization (SMBO) to fine-tune models hyperparameters.

**Figure 4:**
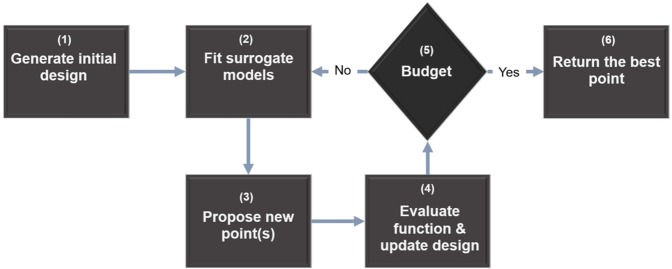
General SMBO approach.

Sequential model-based optimization (Fig. 4) is unique automated approaches for solving algorithms configuration and hyperparameter optimization of expensive black-box models. SMBO is known to converge for the low computational budget performance is due to: (1) the capability to reason about the quality of experiments before they are run; and (2) advancing from the “adaptive capping” to avoid long run^3^.

When it comes to model implementation, only RF and SVM require tuning to some of its hypermeters. The overall-hyperparameters utilized for each model was summarized along with its value, short description, and the package used to run the model, is in Table 2. The search space for each required hyperparameters was set according to guides and manuals of each package that implement each model.

**Table 2:** Parameters set utilized by each model along with respective values.

Model	Package	Parameters	Explanation	Value
RF	“A Fast Implementation of Random Forests ranger” Formerly: “*ranger*” package^63^.	Replace	Sample with replacement	False or True
		respect.unordered.factors	Handling of unordered factor covariates	True (default)
		sample.fraction	Fraction of observations to sample	From 0.632 to 1
		num.trees	Number of trees	From 2^5^ to 2^10^
		mtry	Number of variables	From 2 to 8
SVM	“Misc Functions of the Department of Statistics, Probability Theory Group, TU Wien” Formerly: “*E1071*” package^64^.	Kernel	Kernel function	Radial or polynomial
		Cost	Regularization cost	From 2^−15^ to 2^15^ (default)
		gamma (if kernel =: “radial”)	Kernel width	From 2^−15^ to 2^15^ (default)
		degree (if kernel =: “polynomial”)	Polynomial degree	From 1 to 8 (default)

Only “mtry” and “num.trees” are allowed to fix by the user according to some instructions and strategies. Or else, the left parameters are set exactly to the allowed (or default) values (or range of values) by each package. The number of variables is for each tree (i.e. “mtry”), various heuristics recommended by packages that provided RF are used to set the optimum values (Table 3). These heuristics advise that ranges of 2 to 8 would be excellent for “mtry”. On the other hand, the total number of trees to fit (i.e., “num.trees” for RF) is set to exponential rate via a base of 2 (i.e.2^i^, i = 5, …, 11). By allowing for the instructions of the used packages and some experimental researches, an optimal value of 2^5^ to 2^10^ was set^3^.

**Table 3:** Heuristics proposed by packages instructions to set the optimal number of variables for RF.

package	Suggested Value
	*mtry*
*gbm*	N.A
*ranger*	$\sqrt{{{\rm{N}}}_{{\rm{i}}}}$ = 3
*xgboost*	6
*h2o*	2 to 8
*Random forest*	$\sqrt{{{\rm{N}}}_{{\rm{i}}}}$= 3
**N**_**i**_: Total number of variables (i.e. 12)	

During tuning, hyperparameters need to be carefully optimized, so as much accuracy the model is achieving, the model selection will be reliable. In general, the tuning process must be a formal and quantified part of the model evaluation yet, in most cases personal experience and intuition, heavily intervene by influencing the process in ways that are hard to quantify or describe^46^. In this study, three techniques were implemented, i.e., LR, RF, and SVM, only LR is straight forward and does not require any further tuning. The training process was started by searching the optimal parameters using SMBO with 10-fold cross-validation on the training set that represents 70% of the input data to prevent overfitting. The chosen optimum pairs of hyperparameters that have the highest classification accuracy are shown in Table 4.

**Table 4:** The optimum parameters obtained by the tuning process.

Data Task	Landslide models							
	RF				SVM			
	Replace	sample.fraction	num.trees	mtry	kernel	cost	gamma	degree
*Lidar 5 Meters*	FALSE	0.906	100	3	radial	2^0.843^	2^−2.667^	N/A
*Lidar 30 Meters*	FALSE	0.866	49	6	radial	2^1.517^	2^0.224^	N/A
*ASTER 30 Meters*	FALSE	0.969	164	2	radial	2^5.619^	2^−3.048^	N/A

### Models evaluation and comparison

Various performance metrics can be executed for quantitative assessment; however, we consider the Accuracy (Acc) as main metric for hyperparameters tuning and one of the main overall performance indicator metrics for the landslide predictive models. In this study, Acc together with Cohen kappa index (kappa)^47^ and the Area under the ROC Curve (AUC), were used to evaluate the overall performance and the predictive capabilities of the tuned models.

Additionally, model performance was evaluated using one of the most important non-parametric tests called the Friedman test^48^. The Friedman test is heavily used for multiple comparisons to perceive significant differences between the performances of two or more approaches because the test involves no previous information for the used data and still is valid even if the data are normally distributed and was designated in this study^49^.

The Friedman test has a null hypothesis, viz., there are no differences between the performances of the landslide models. The *p*-value is the probability of refusing the null hypothesis if the hypothesis is true. Then each model is assessed. The higher the p-value, the more likely that the null hypothesis is rejected.

Another useful use for the Friedman non-parametric test is the ability to obtaining a “Critical differences” diagram of multiple classifiers. A value called “Critical Difference” (i.e. calculated according to the equation below) indicates the critical average rank performance. If the average rank of the classifiers is within the critical difference distance (CD) then they are not statistically significantly different.

7 $${{\rm{CD}}}_{{\rm{\alpha }},{\rm{K}},{\rm{N}}}\approx {{\rm{q}}}_{{\rm{\alpha }},{\rm{K}}}\sqrt{\frac{{\rm{K}}({\rm{K}}+1)}{6{\rm{N}}}}$$

where: *α* is the confidence level, *K* is the number of models and N is the number of measurements. To calculate q_*α*, K_ the Studentised range statistic for infinite degrees of freedom divided by $\,\sqrt{2}$ is used.

### Landslide susceptibility map assessments

At the end of the validation and assessment processes, landslide susceptibility maps can be generated to: (1) assess the quality of the generated maps; and (2) check the input dataset for its suitability for later usage in other tasks (i.e., decision making) because it’s common to have some variables that have high correlation or even multicollinearity and these variables need to be check before using them as variables. However, to achieve those goals a key step must be performed. Usually, that step involves assessing the sufficiency and accuracy of the generated susceptibility maps based on the empirical assumption that state: “A model is sufficient and accurate when there is an increasing landslide density ratio when moving from low to high susceptible classes and high susceptibility classes cover small areas extent^3,9^”. This means, a sufficiency analysis is essentially based on susceptibility maps and can be implemented by: (i) reclassifying the probability pixels produced for the whole study area by each model; (ii) overlying the existing landslide inventory over the susceptibility maps so to be able to obtain representative statistic for each susceptibility class (i.e., landslide density and extent).

## Scale Effects of Geomorphometric Factors

**Figure 5:**
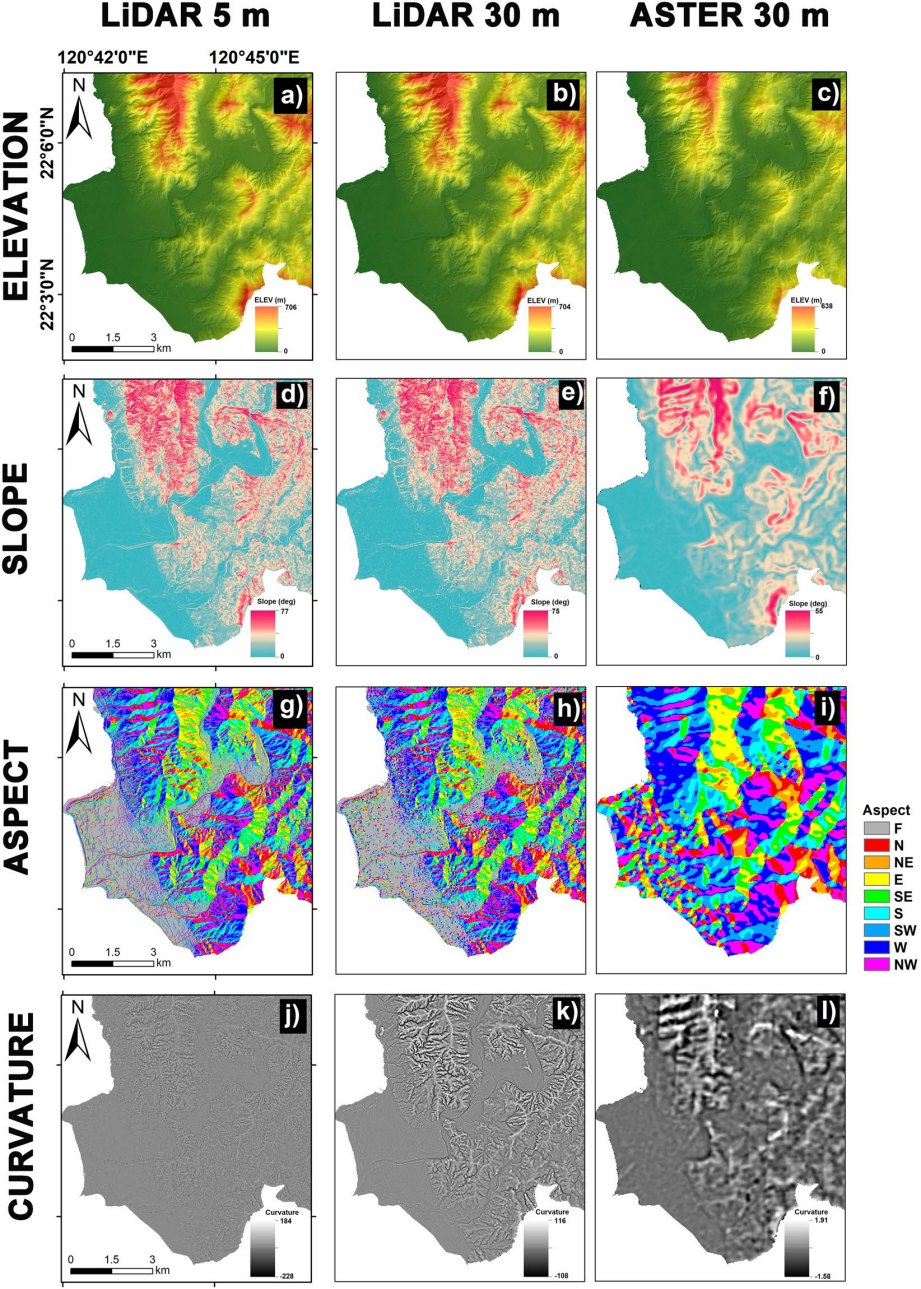
The used DEMs in different resolution and their corresponding geo-morphometric factors: (**a**,**b**,**c**) elevation, (**d**,**e**,**f**) slope, (**g**,**h**,**i**) aspect, (**j**,**k**,**l**) total curvature.

**Figure 6:**
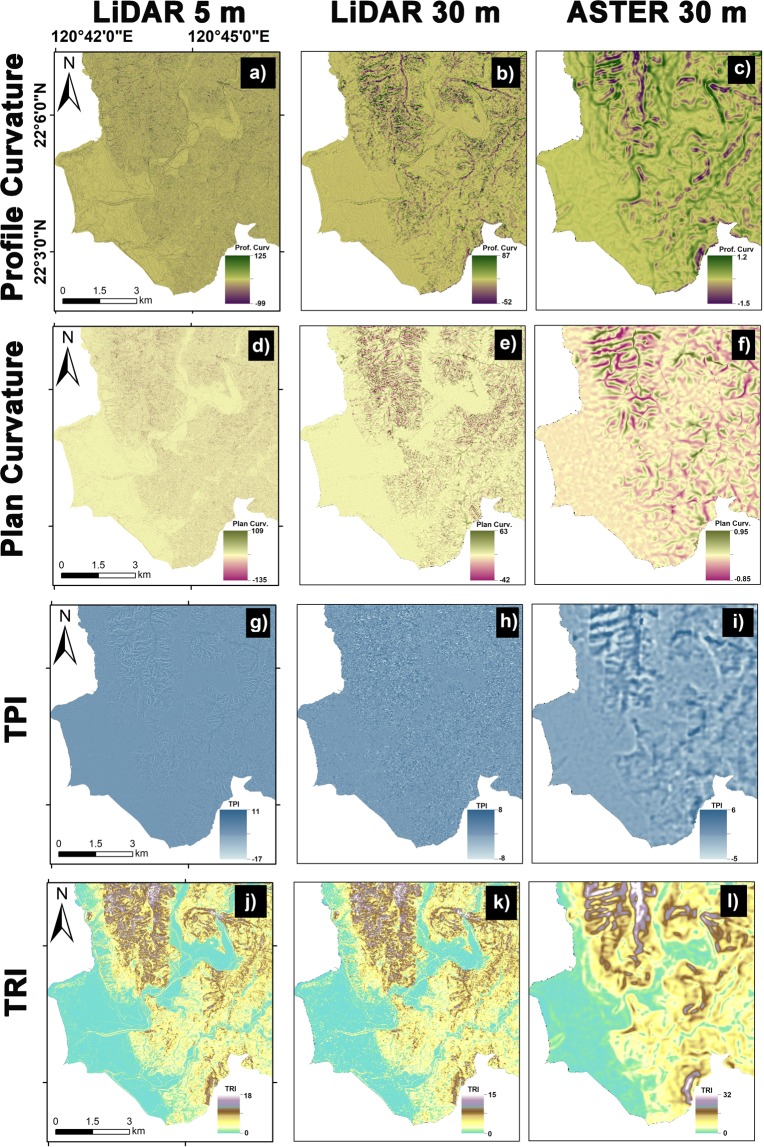
The used DEMs in different resolution and derived corresponding geo-morphometric factors: (**a,b,c**) profile curvature, (**d,e,f**) plan curvature, (**g,h,i**) Topographic Position Index (TPI), and (**j,k,l**) Topographic Ruggedness Index (TRI) for 5 m Lidar DEM, 30 m Lidar DEM, and 30 m ASTER DEM, respectively.

It has been proved in the literature that topographic variables coming from a digital elevation model are the prime component for any susceptibility analysis. Furthermore, several studies indicated that the quality of DTMs would affect the overall model results^27,30^. Therefore, the certainty factor (CF) method had been conducted to analyze the scale effects of geo-morphometric factors for two kinds of DEMs in different quality and resolution in this section. For this, the 5 m Lidar DEM is downsampled to 30 meters to have a comparison with ASTER DEM, and then the CF values are calculated according to the landslide characteristics in each geo-morphometric factors generated by the three elevation models. The 5 m Lidar DEM, 30 m Lidar DEM, 30 m ASTER DEM, and their derivative factors for the study area is shown in Fig. 5 and 6(a–l), respectively. Subsequently, these DEMs were employed to produce the LSM maps.

## Results and Analysis

### The relationship between landslide causative factors and landslide occurrence

The relationship between landslide causative factors and landslide occurrence was identified by CF model using 5 m Lidar DEM as shown in Table 5. This table presents the CF value for all causative factors, including the eight geo-morphometric factors. Additionally, the CF value statistics of eight geo-morphometric factors with the varying resolution of DEMs (5 m LiDAR, 30 m LiDAR and 30 m ASTER DEM) are summarized in Table 6 for the exploration of the scale effects. According to the distribution of the CF value in Table 5, for the higher Elevation, Slope, TRI, and TPI values, the certainty increased in landslide occurrence. With Aspect value of 66–247°, i.e., slope face to northeast-southwest direction, the certainty increased in landslide occurrence. No matter the type of curvature, the larger or smaller its value, the larger its corresponding CF value. For lithology, Type II, V, and VI corresponds with a positive CF value, i.e., the certainty increased for the shale and thin alternation of sandstone and shale with thick-bedded sandstone and conglomerate lentil (Type II), thick-bedded sandstone, interbedded sandstone and shale (Type V), and thick-bedded sandstone intercalated with conglomerate (Type VI). The thick-bedded sandstone intercalated with conglomerate has the least cementation degree and the highest material discontinuity. Therefore, the inter-layer slip is most likely to occur. Besides, the closer to the drainage networks, the greater the landslide occurrence.

**Table 5:** The CF computation result of each causative factor with value ranges for the 5 m Lidar DEM.

Factors	Class	Percent of area (%)	No. of landslide pixel	Percent of landslides (%)	PPa	CF
Elevation (m)	0~63	39.935	2934	9.219	0.003	−0.767
	63~145	27.257	5933	18.641	0.008	−0.313
	145~242	20.276	9857	30.971	0.019	0.341
	242~383	10.159	9149	28.746	0.035	0.639
	383~706	2.361	3954	12.423	0.065	0.800
Slope (^o^)	0~15	50.521	2108	6.623	0.002	−0.867
	15~20	13.858	814	2.558	0.002	−0.813
	20~25	11.292	1144	3.594	0.004	−0.679
	25~30	8.097	1894	5.951	0.009	−0.262
	30~40	11.549	9815	30.839	0.033	0.618
	40~78	4.671	16052	50.435	0.134	0.896
Aspect	0~66	30.394	3158	9.922	0.004	−0.671
	66~128	10.768	6663	20.935	0.024	0.480
	128~190	13.617	13153	41.327	0.038	0.662
	190~247	15.463	5936	18.651	0.015	0.169
	247~302	16.889	1504	4.726	0.003	−0.718
	302~360	12.857	1413	4.440	0.004	−0.652
TRI	0~1	51.686	1939	6.092	0.001	−0.881
	1~2	28.488	2702	8.490	0.004	−0.699
	2~3	13.490	8641	27.150	0.025	0.497
	3~4	4.796	11641	36.576	0.095	0.858
	4~5	1.010	5065	15.914	0.196	0.925
	5~19	0.303	1839	5.778	0.237	0.936
TPI	(−18)~(−2)	0.255	382	1.200	0.059	0.778
	(−2)~(−1)	1.827	1903	5.979	0.041	0.686
	(−1)~0	61.735	15183	47.705	0.010	−0.225
	0~1	34.788	12628	39.677	0.014	0.122
	1~2	1.088	1454	4.568	0.052	0.753
	2~12	0.080	277	0.870	0.135	0.896
Total curvature	<(−28)	0.231	326	1.024	0.055	0.765
	(−28)~(−12)	3.862	2997	9.417	0.030	0.583
	(−12)~(−4)	25.352	8766	27.543	0.014	0.079
	(−4)~0	40.479	7324	23.012	0.007	−0.428
	0~8	26.008	9088	28.554	0.014	0.089
	>8	4.058	3326	10.450	0.032	0.604
Profile curvature	<(−6)	3.317	2483	7.802	0.029	0.568
	(−6)~(−2)	20.748	7353	23.103	0.014	0.101
	(−2)~2	52.287	12175	38.254	0.009	−0.265
	2~6	20.327	7082	22.252	0.014	0.086
	6~13	2.942	2238	7.032	0.030	0.575
	>13	0.367	496	1.558	0.053	0.755
Plan curvature	<(−14)	0.314	461	1.448	0.057	0.774
	(−14)~(−6)	2.633	2171	6.821	0.032	0.607
	(−6)~0	69.607	15452	48.550	0.009	−0.299
	0~6	24.559	10885	34.201	0.017	0.279
	6~16	2.801	2565	8.059	0.036	0.645
	>16	0.075	293	0.921	0.153	0.907
Annual average rainfall (mm)	0~2745	0.01	0	0.00	0.000	−1.000
	2745~2922	10.44	0	0.00	0.000	−1.000
	2922~3066	19.63	1802	5.66	0.004	−0.709
	3066~3268	49.63	29389	92.34	0.023	0.457
	3268~3609	20.260	636	2.00	0.001	−0.900
Lithology	Type I	38.570	1566	4.92	0.002	−0.871
	Type II	37.110	13675	42.97	0.014	0.135
	Type III	5.320	1082	3.40	0.00794	−0.358
	Type IV	12.05	805	2.53	0.003	−0.788
	Type V	1.170	614	1.93	0.020	0.388
	Type VI	5.780	14085	44.25	0.095	0.859
Distance from road (m)	0~50	20.920	1280	4.020	0.002	−0.806
	50~100	14.280	1858	5.840	0.005	−0.588
	100~150	10.190	1374	4.320	0.005	−0.573
	150~200	7.400	1467	4.610	0.008	−0.373
	>200	47.210	25848	81.210	0.021	0.414
Distance from drainage networks (m)	0~50	16.440	9755	30.650	0.023	0.458
	50~100	13.570	6803	21.370	0.020	0.361
	100~150	12.120	3966	12.460	0.013	0.028
	150~200	11.150	3130	9.830	0.011	−0.116
	>200	46.710	8173	25.68	0.007	−0.447

**Table 6:** The CF results of eight geo-morphometric factors with different quality and resolution of DEMs.

Factors	Range	CF Values		
		Lidar DEM		ASTER DEM
		5 m	30 m	30 m
Elevation	0~63	−0.767	−0.783	−0.799
	63~145	−0.313	−0.252	−0.360
	145~242	0.341	0.346	0.275
	242~383	0.639	0.630	0.696
	383~706	0.800	0.789	0.797
Slope (^o^)	0~15	−0.867	−0.872	−0.408
	15~20	−0.813	−0.808	−0.238
	20~25	−0.679	−0.503	0.174
	25~30	−0.262	−0.296	0.322
	30~40	0.618	0.625	0.837
	40~78	0.896	0.892	0.847
Aspect	0~66	−0.671	−0.657	−0.376
	66~128	0.480	0.525	0.353
	128~190	0.662	0.636	0.499
	190~247	0.169	0.124	0.208
	247~302	−0.718	−0.672	−0.536
	302~360	−0.652	−0.554	−0.562
TRI	0~1	−0.881	−0.888	−0.930
	1~2	−0.699	−0.654	−0.958
	2~3	0.497	0.518	−0.677
	3~4	0.858	0.852	−0.721
	4~5	0.925	0.922	−0.543
	5~19	0.936	0.934	0.584
TPI	−18~−2	0.778	0.879	0.951
	−2~−1	0.686	0.743	0.771
	(−1)~0	−0.225	−0.291	−0.466
	0~1	0.122	0.149	−0.270
	1~2	0.753	0.768	0.466
	2~12	0.896	0.877	0.955
Total curvature	<−28	0.765	0.853	−1
	−28~−12	0.583	0.623	−1
	−12~−4	0.079	0.034	−1
	−4~0	−0.428	−0.468	0.043
	0~8	0.089	0.119	−0.055
	>8	0.604	0.606	−1
Profile curvature	<−6	0.568	0.620	−1
	−6~−2	0.101	0.116	−1
	−2~2	−0.265	−0.284	0.00001
	2~6	0.086	−0.019	−1
	6~13	0.575	0.643	−1
	>13	0.755	0.790	−1
Plan curvature	<−14	0.774	0.821	−1
	−14~−6	0.607	0.650	−1
	−6~0	−0.299	−0.326	0.139
	0~6	0.279	0.318	−0.149
	6~16	0.645	0.566	−1
	>16	0.907	0.906	−1

From Table 6, it is observed that different spatial resolutions of topographic data do not affect largely on the trend of CF values, except for the impact on the curvature factors, comprising total, profile, and plan curvature because curvatures are defined by means of a second derivative of the elevation and a second derivative amplifies greatly even the smallest differences between the DTMs. However, the results on different data quality of DEMs indicate that geomorphometric features cannot be accurately derived from a lower data quality of DEM, e.g., an ASTER DEM. Therefore, the CF values shown a different trend for ASTER DEM compared with ones derived from Lidar DEM with the same resolution. For example, CF values with Slope of 20–30° changed from negative to positive. And ones with TRI of 2–4 changed from positive to negative. Moreover, the CF value of −1 on curvature factors shows that results with a lower data quality of ASTER DEM are unable to render more detailed topographic curvature. It implies that some geomorphometric features derived from different data quality of DEMs will be affected significantly on the landslide susceptibility modeling.

### Landslide susceptibility map assessments

**Figure 7:**
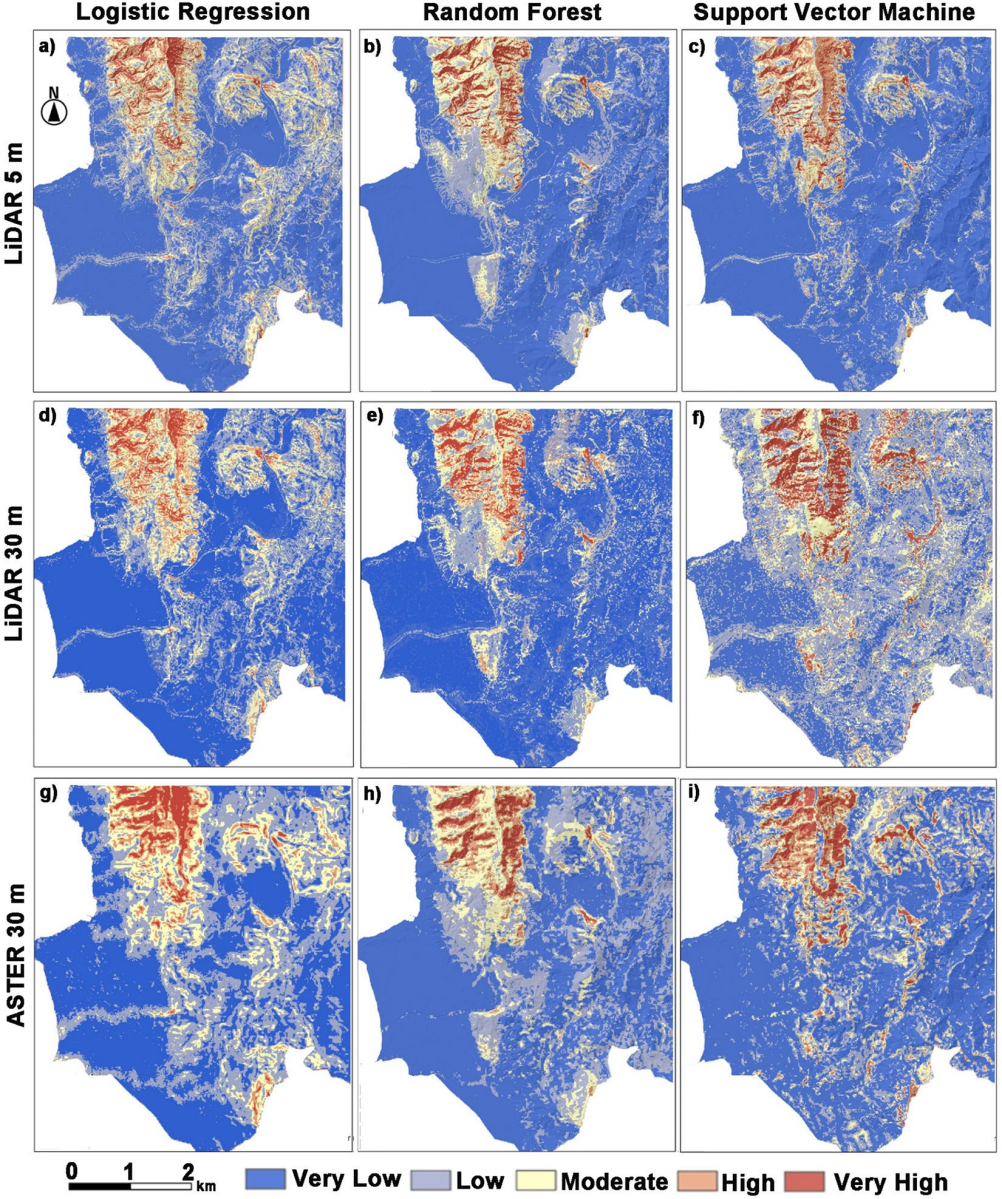
LSM maps produced by LR, RF, and SVM models, respectively, using different resolution DEMs.

**Figure 8:**
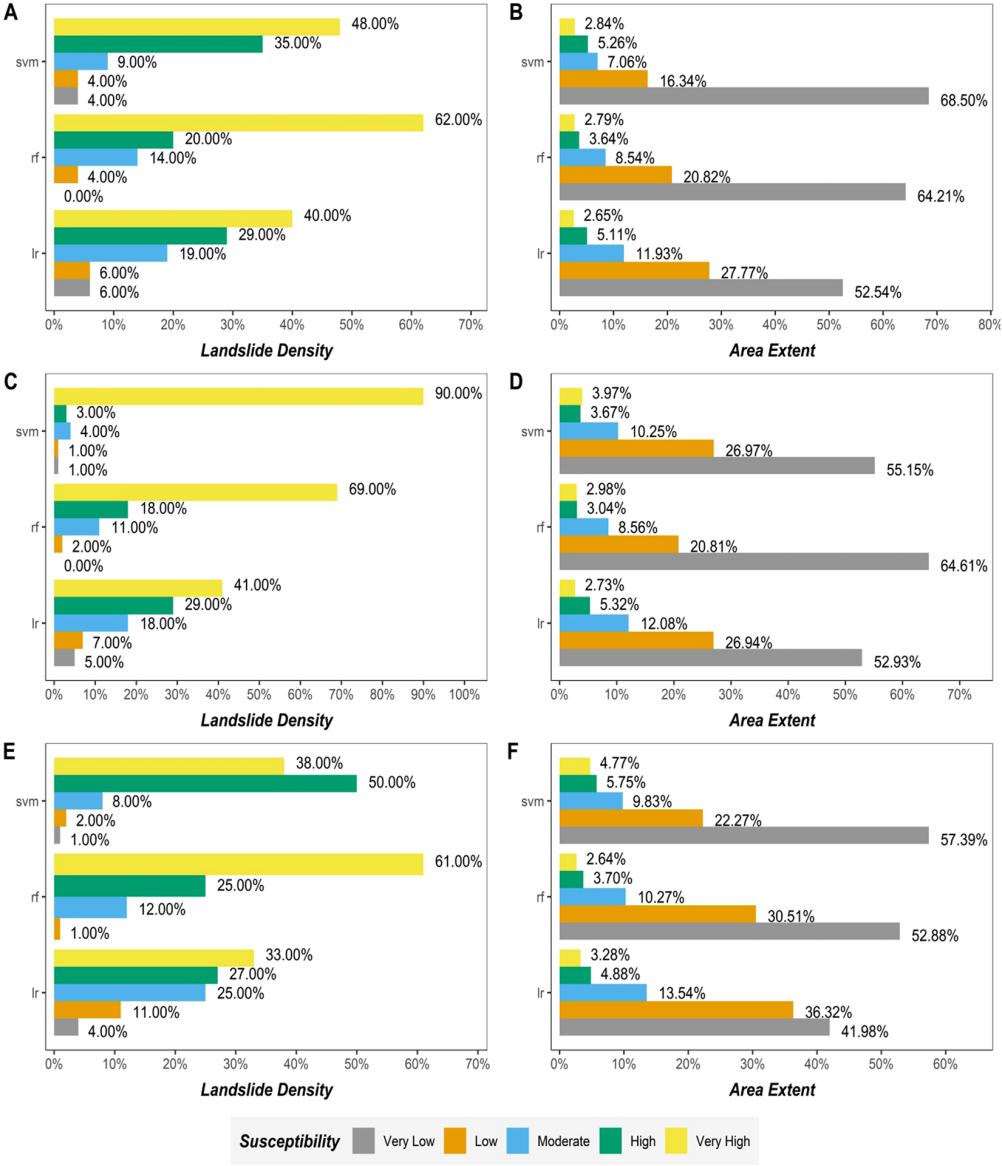
Sufficiency analysis of the landslide susceptibility maps: (Left) Landslide density distribution by susceptibility zones; (Right) Total area covered by susceptibility zones; (**A**,**B**) Lidar 5 meters, (**C**,**D**) Lidar 30 meters; (**E**,**F**) ASTER 30 meters; (Left).

Achieving decent models’ performance is not the end road for LSM analysis. Additional steps involve:(1) creating the landslide susceptibility maps for the case study area in the form of probability grids using the validated models; (2) reclassifying susceptibility grids; and (3) analyzing the overall grids and assess its quality. The initial two steps are based on predicting the study area probabilities toward landsliding and afterward, a simple reclassification into five susceptibly classes that vary from very low to very high (Fig. 7) using Table 7 is performed. The last step is critical for understanding the overall pattern of landslides distribution and landslides susceptible areas and can be performed by attaining a landslide density distribution by overlapping the existing inventory map over the generated susceptibility maps and afterward, a summary statistic for the area covered by each susceptibility classes (Fig. 8) is obtained.

**Table 7:** The probability intervals classes used to classify the landslide susceptibility maps.

*Susceptibility Class*	*Very Low*	*Low*	*Moderate*	*High*	*Very High*
*Probability Range*	0 ~ 0.15	0.15 ~ 0.45	0.45 ~ 0.75	0.75 ~ 0.90	0.90 ~ 1

A visual analysis of the resulting LSM maps (Fig. 7), shows a smooth surface produced by each model for each DEM dataset. An obvious differentiation between Lidar datasets (5 and 30 meters) maps and ASTER dataset maps is represented in the form of very smooth transitioning from each susceptibility class to another. The results of sufficiency analysis (Fig. 8) were positive as they fulfilled the two required spatial conditions: (1) landslide pixels should belong to the highest susceptible class available; and (2) the extent areas covered by higher susceptible classes need be lower as possible. The results are similar to models evaluation results, LiDAR datasets (i.e., 5 and 30 meters) in particular and RF models, in general, achieve better results than the rest of the models with well-balanced outcomes that put confidence in the overall LSM produced by either Lidar 5 meters or 30 meters. However, it is very crucial to understand that landslide density in Fig. 7a,c,e have a moderate presence of landslide events in very low susceptibility class despite the models achieved excellent scores regarding performance metric and that is due to how the stable non-landslide samples are sampled. Usually, misclassifications on the extremes (very low and very high) tend to indicate the overall confidence in the misclassification of the model, but that depends on modeling experiment conditions.

### Model evaluation and comparison

The optimum hyper-parameters obtained in Table 8 for each model in each respective dataset, were used to train each model and assess the overall performance of the models using performance metrics indicators such Acc, AUC, and Kappa index.

**Table 8:** Overall performances of the tuned models.

Data Task	Model	Metric					
		Test stage			Train stage		
		*Auc*	*Acc*	*Kappa*	*Auc*	*Acc*	*Kappa*
*Lidar 5 Meters*	*LR*	0.885	0.807	0.613	0.917	0.854	0.707
	*RF*	0.935	0.868	0.737	0.999	0.986	0.972
	*SVM*	0.905	0.856	0.712	0.967	0.928	0.855
*Lidar 30 Meters*	*LR*	0.916	0.860	0.720	0.904	0.841	0.683
	*RF*	0.966	0.893	0.786	1.000	0.991	0.982
	*SVM*	0.913	0.848	0.697	1.000	0.995	0.989
*ASTER 30 Meters*	*LR*	0.854	0.786	0.573	0.904	0.831	0.661
	*RF*	0.881	0.844	0.687	0.997	0.974	0.947
	*SVM*	0.929	0.881	0.761	1.000	0.997	0.993

**Figure 9:**
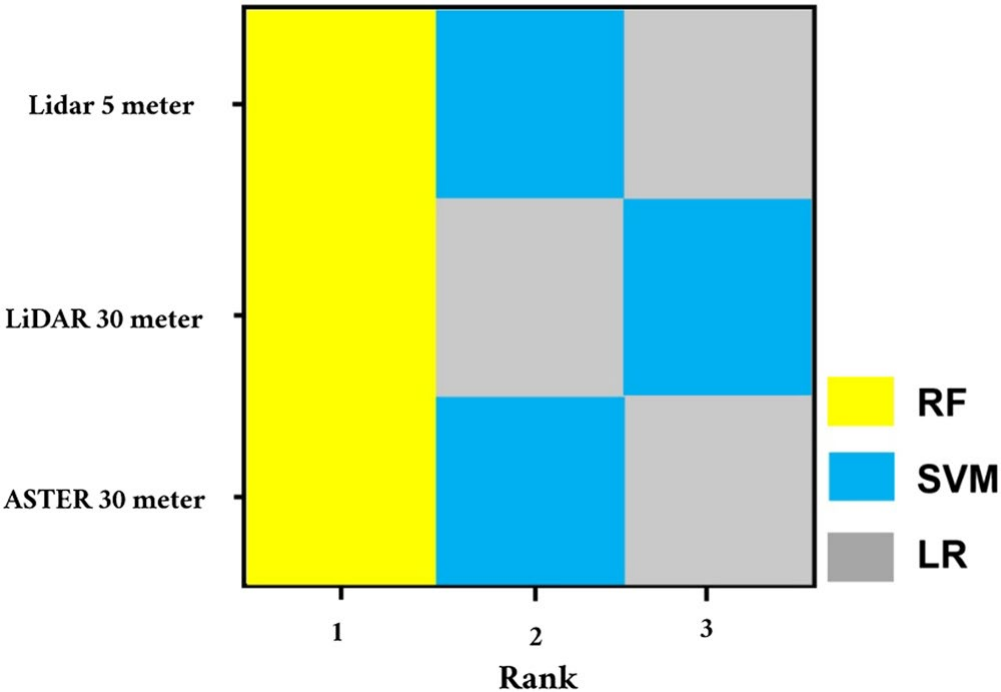
The overall rank matrix of the implemented models based on performance results.

**Figure 10:**
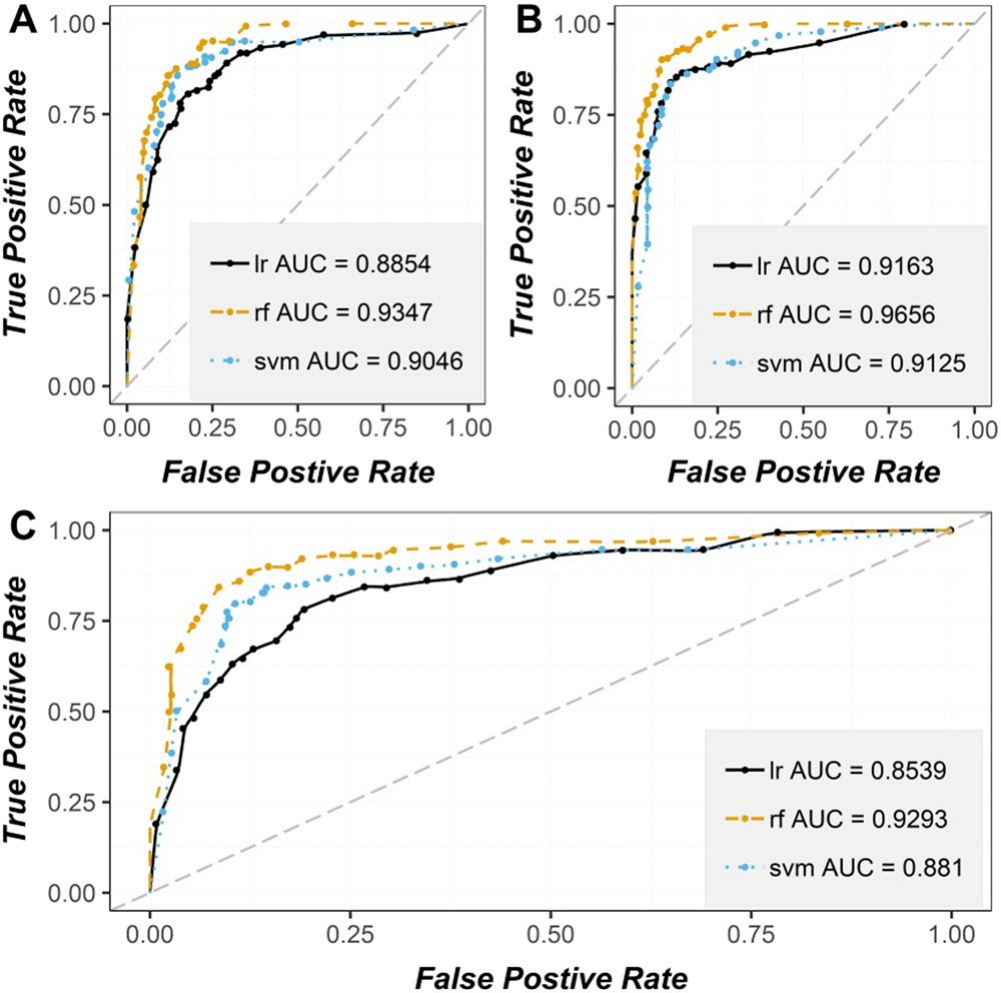
Stacked ROC curves of the implemented models: (**A**) Lidar 5 meters; (**B**) Lidar 30 meters; and (**C**) Aster 30 meters.

The generated overall rank matrix of the implemented models (Fig. 9) based on performance results (Table 8 and Fig. 10) are generally in favor of RF being ranking top of all model in all datasets, followed up by either SVM or LR depending on the dataset for (i.e. LR on Lidar 30 meters dataset was able to achieve better results than SVM). However, a detailed analysis on the dataset level shows that Lidar 5 meters dataset models achieved far better results than Aster 30 meters dataset models, but surprisingly the highest performance results in term of all metrics were achieved by the resampled Lidar Dataset from 5 meters to 30 meters. These dataset models were able to achieve excellent results exceeding closest dataset models (i.e., Lidar 5 meters) by a margin ranging from 1% to 3%, 1% to 1.5% and 4% to 10% in term of AUC, Acc and Kappa respectively.

Despite the fact, the difference between each dataset models regarding performance results is relatively noticeable. However, Friedman non-parametric test at the significant level α = 5%was performed on models’ performance results in all datasets rather than inside each dataset (Table 9). These results show that the differences in performance between the implemented model are statistically insignificant between datasets because the *p value *exceeds the significant level of 0.05.

**Table 9:** Friedman rank test results.

Degree of Freedom	chi–squared	*p* value	Significance
2	4.667	0.097	No

**Figure 11:**
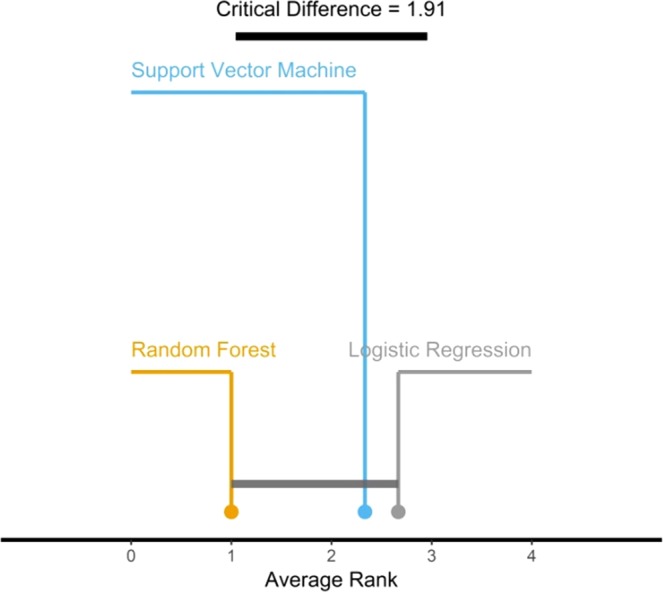
Critical difference plot of the implemented models; Values on the top indicate average rank performance (i.e., 1.91).

Additionally, the critical difference plot (Fig. 11) generated using the Friedman non-parametric test, shows that there’s a line connecting models indicating that they are within the insignificance range (i.e., critical difference range) of 1.91, which means that there are no statistical differences among all model.

## Discussion

### Effect of grid resolution and data quality on susceptibility models

Landslide susceptibility assessment is a useful task for landslide hazard management and mitigation^8,9,50^. However, landslide is a complex natural phenomenon which is controlled by several geo-environmental factors; thus, it is not easy to be modeled accurately^8,9^. Data-driven models are proved to be an effective tool for landslide susceptibility modeling^19,29^. Very recently, a large number of machine learning approaches are adopted and applied successfully for landslide susceptibility assessment^8,21^. However, the performance of these models depends mainly on the input data. Therefore, it is essential to test and check the quality of the data before providing it as an input in the learning models. Typically, a large portion of the input factors in susceptibility modeling comes from a DEM^8^. Consequently, the quality of DEM data or more specifically, the DEM derived causative factors used in the model are very crucial input for producing an accurate LSM output. In addition to having an appropriate quality, the scale of selected DEM is also vital in landslide hazard assessment. This is because the details of the topographic information provided in a DEM depends upon its spatial resolution^29,51^. Several studies consider the DEM resolution as a first filter that assimilated into a model^52,53^. Researchers are often direct for the highest spatial resolution product for mapping the finest details^13^. However, an increase in spatial resolution means increased computational requirements for pre-processing the data. Moreover, with different DEM resolution, the primary topographic attributes such as slope angle and curvature exhibit substantial local variations^54^.

In this study, we have demonstrated the scale effects of geomorphometric factors derived from two DEMs with varying spatial resolutions (i.e., LiDAR and ASTER) in analyzing the landslide susceptibility of Sihjhong watershed region. Contrary to the general expectations, but in line with the findings of Catani *et al*.^31^ and others^27,29,30^, our result shows that a fine raster resolution DEM (5 m) does not significantly help in increasing the model prediction accuracy. Accuracies (AUC Values – see Fig. 9) obtained for the three different data-driven models indicate that 30 m resampled LiDAR DEM produces the best fit with the field data. Probable reasons are highlighted below why a finer MUR does not necessarily provide the best results. Firstly, landslide susceptibility assessments are dealing with the local geomorphological processes. Like any other geomorphic processes, landslides are also influenced by the morphology measured at the mesoscale level that is more representative of the hillslope forms and processes of such kind. However, finer DEMs would account for topography variations at the micro-scale, and probably those forms are not very much related to mesoscale processes like landslides.

Furthermore, the minimum landslide size mapped from the satellite images is 0.1 hectare, hence the LSM results from a 30 m resolution DEM is a good option. Excessive detailing of topography from the high-resolution models are discussed in several studies and pointed out that the general trend of relief is often a better predictor of mesoscale processes than detailed information^55,56^. Additionally, slope and curvature derived from a fine resolution DEM are higher than the coarser resolutions (see Fig. 5); this may result in more number of false positive rates. Similar results were also noticed in other studies^29,53,57^. Zhang and Montgomery^51^, portrayed that for many landscapes, a medium resolution grid size explores a rational compromise between improving resolution and data volume for simulating geomorphic and hydrological processes. Therefore, appropriate DEM resolution should be selected depending upon the aim of the modeling, characteristics of the study area, and the availability of data.

On the other hand, sub-par quality of DEM can decrease the modeling accuracy as well. Therefore, the CF values showed a different trend for 30 m ASTER DEM compared with the one derived from Lidar DEM with exactly the same resolution. Although the terrain representation by ASTER GDEM used in this study is superior to SRTM‐3 for most landform elements^58^, their accuracies for forested terrains and low elevated regions remains questionable^59,60^. Furthermore, when compared with locally derived LiDAR DEMs, their RMSE is found to be large^60^. This implies that ASTER DEM has inherent artifacts in producing a realistic representation of terrain features. A large part of inherency comes from the processing stage itself as they were developed from a compilation of over 1.2 million ASTER AVNIR scenes, many of it contains clouds obscuring the features. The aforementioned artifacts in ASTER DEM will also inherent to their derivatives^61^.

### Execution time of different susceptibility assessments

A random sampling of non-landslides points from the overall study area carry some artifacts and randomness to the evaluation process and that randomness can vary in size and effect. This drawback is one of the disadvantages of LSM using ML modeling, and efficiently eliminating those artifacts and randomness is nearly impossible. To overcome such drawback, machine learning needs to have decent performance with less computational time (i.e., execution time).

**Figure 12:**
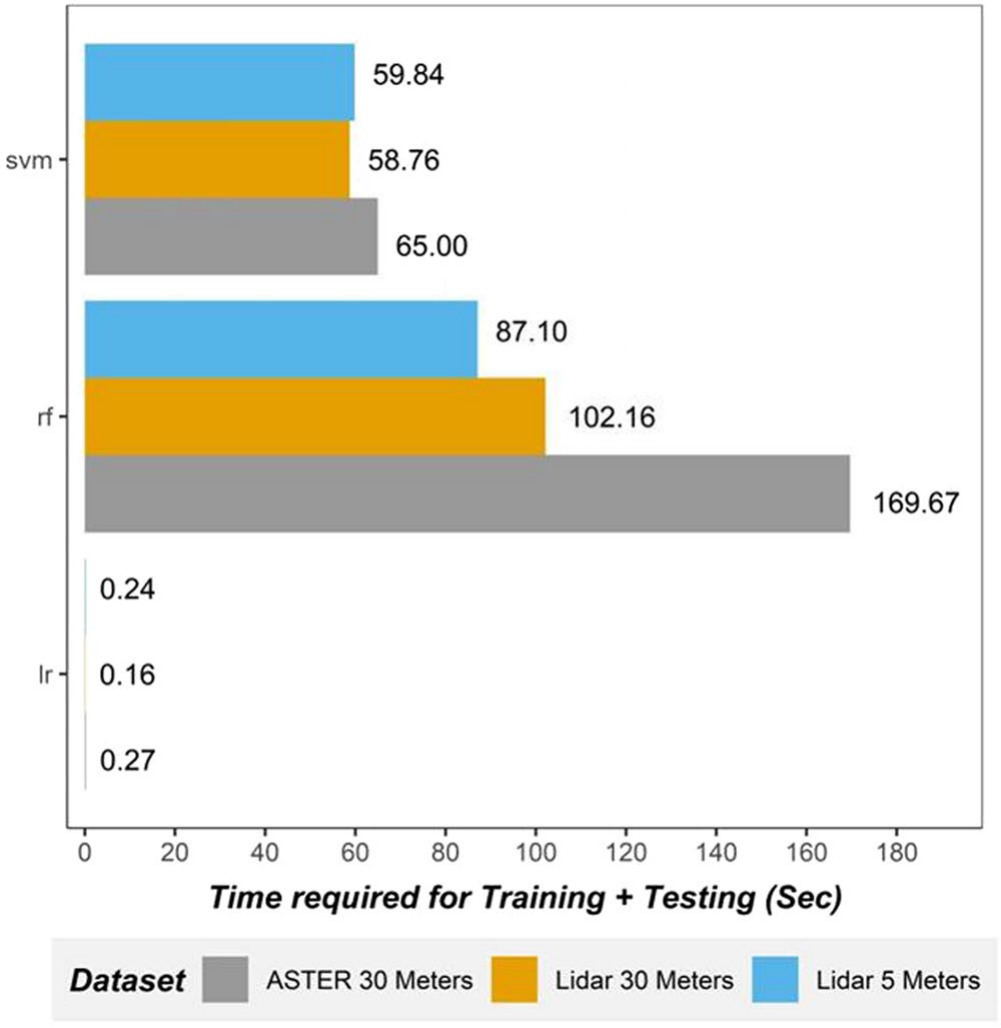
Comparison of the average time* (in seconds) required to training and testing each model in each dataset (*excluding the hyperparameters tuning time).

The results of computational time required for each model (training and testing the final models excluding the time spent on tuning the hyperparameters) and each dataset (Fig. 12) shows that SVM models are at least 50% faster than RF, and LR models are 50 times faster than SVM. Besides, 5 meters Lidar DEM based models required a relatively close computational time to LSM models based of 30 meters (i.e., LR and SVM), except for RF and LiDAR 30 meters dataset slightly require less computational time for LR and SVM models compared to the result of datasets. Note that the pre-processing time for deriving topographical variables from 5 m DEM is much larger than the 30 m DEMs. Therefore, the overall performance results that reported in Tables 8, 9 and Figs 8–12, when combined with the computational process, it is obvious that resampling the LiDAR dataset (i.e. from the original 5 meters to 30 meters) with LR and/or RF models combination would be “Go To” solution as they provide decent results. However, it is widely accepted that no single or particular model can be depicted as the most suitable for all case scenarios, as it depends on the subjective opinion of the decision-maker of whether the more accurate results matter more than the computational time or vice-versa. After all, recent studies^13,62^ suggest that a rather fast and simple model, such as SVM would be much better than an advanced machine learning models like RF, if the consideration was not solely based on the overall performance but on balance of overall performance and the computational time. For instance, SVMs are useful non-linear classifiers whose goal is not only to classify landslide instances correctly but also to keep the distance between instances and keep the separation of the hyperplane at a maximum. On the other hand, RF models offer an excellent performance with decent interpretability and moderate number of hyperparameters to tune in but require a considerable time budget (they require a lot of time to converge especially if used on large-scale analyses) compared to LR models which are the opposite of being simple, fast, easy to implement, and only able to capture the linear relationship between the causative factors and the landslide susceptibility which translate into poor performance. This makes SVM models appealing for susceptibility evaluation considering the number of hyperparameters to tune in. However, if those hyperparameters are inappropriately set, SVM will often lead to unsatisfactory results^3,13^. Though the computational performance for all the models in this study was quick (i.e., <3 minutes), the aforementioned analysis and discussions will be helpful while dealing with a larger amount of data in the machine learning environment.

## Summary and Conclusions

This paper conducts the scale dependency of DEM data in the analysis of landslide susceptibilities. The study area is characterized by steep slopes with frequent debris flows and landslides in the typhoon seasons. The LiDAR DEMs provided unprecedented high-quality terrain data for detailed topographic representations. This study tested the appropriateness of such high accurate grid sizes in the susceptibility studies. The obtained results highlight that a fine resolution DEM not necessarily produce an accurate LSM as they found to be carrying excessive information. These results are in line with the findings of some previous studies^29–31^. The results prove that entailing different DEM scales introduced different results for the same models. A 30-meter resolution DEM depicting accurate topography could be plausible for LSM as they produced decent levels of generalization of the topography. In fact, higher resolution DEMs introduce more noise, which makes the model perform worse than it supposed to be. Entailing high-resolution DEMs (5 meters Lidar) have proven to be hindered on susceptibility models as they feed a steady flow of data 36 times more than 30 meters DEMs which are supposed to theoretically produce better models. However, in reality, the data flow was treated as noise that worsens the overall resulting models instead of enhancing it, which prove that a generalized DEMs of 30 meters used for DEM-derived condition factors is much valuable than their 5 meters counterpart. Additionally, inappropriate spatial resolution increases the pre-processing time. For this reason, it is suggested that an analysis should be performed to understand the scale effects of topographic variables on landslide susceptibility mapping. Our results also indicate that the scale effects of topographic variables are mainly caused by the resolution impact on topographic parameter derivation, while factors such as geology and rainfall are insensitive to resolutions. For susceptibility mapping, RF models are found to be the best model in term of performance for the study area, while SVM is more suitable in the decision-making process when looking for a balanced LSM model between computational time and overall performance.

Further research is required to test variation over a more continuous range of resolutions (e.g. 10 m, 15 m, and 20 m) in more case studies for reducing some uncertainties behind the obtained results. Also, to enhance the results, deep learning techniques such as convolutional neural network and testing other machine learning models are recommended. The obtained landslide susceptibility maps are based on present and past landslides. However, Future landslides are not foreseeable, and thus the obtained LSM models are obsolete after a given period of time. Thus, the inventory and model should be updated constantly.
